# Prevalence and treatment outcome of bronchopleural fistula: a multi-center study in Ethiopia

**DOI:** 10.1186/s13019-023-02325-y

**Published:** 2023-07-12

**Authors:** Samuel Tesfaye Woldemariam, Israel Bekele Molla, Seyoum Kassa Merine, Dereje Gulilat Yilma

**Affiliations:** 1 Department of Surgery, School of Medicine, Institute of Health, Jimma University, Jimma, Ethiopia; 2 School of Nursing, Institute of Health, Jimma University, Jimma, Ethiopia; 3 Department of Surgery, School of Medicine, College of Health Sciences, Addis Ababa University, Addis Ababa, Ethiopia

**Keywords:** Bronchopleural fistula, Treatment outcome, Ethiopia

## Abstract

**Purpose:**

The study aimed to assess the magnitude, risk factors, and management outcome of patients with a bronchopleural fistula at multiple centres in Ethiopia.

**Method and materials:**

A ten years (September 2012 - August 2021) institution-based multicenter retrospective cohort study was conducted from September 13 to September 30, 2021. we surveyed the cards of all patients having a diagnosis of bronchopleural fistula for the last 10 years. The document was reviewed using an extraction checklist. Descriptive statistics (mean, standard deviation, frequency, percentages) and crosstabulation were used to describe the outcome variable.

**Result:**

A total of 52(2%) patients were diagnosed to have bronchopleural fistula out of 2546 patients admitted to the cardiothoracic unit in three hospitals from September 2012 - August 2021 and 69% of study participants were male. The mean age of study participants was 33.42 years with SD = 12.5. Thirty-one (60%) of the cases spontaneously developed a bronchopleural fistula and 20 (38%) were post-surgical and 1(2%) was a post-traumatic fistula. Of the total of post-surgical bronchopleural fistula, 14 (26.9%) of them were lung resection, 4 (7.7%) were hydatid cystectomy and 1(1.9%) are decortications, and bullectomy respectively. of the total post-lung resection, 8 (57%) were pneumonectomies followed by 3 (21.5%) Lobectomy, 2 (14.5%) wedge resection and 1(7%) bilobectomy respectively. Fifty patients were managed surgically and two patients were managed conservatively. Bronchopleural fistula (BPF) was closed in 40 (85.4%) and there were two (3.9%) deaths, and the cause of death was sepsis secondary to pneumonia of the contralateral lung in one case.

**Conclusion:**

Having thoracic surgery is a risk factor for the development of bronchopleural fistula. Management of bronchopleural fistula needs to be individualized.

## Introduction

Bronchopleural fistula (BPF) is a sinus tract between the bronchus and the pleural space that may result from necrotizing pneumonia, lung neoplasm, blunt and penetrating lung injuries, may occur as a complication of procedures or may complicate radiation therapy [1, 2]. It is one of the serious complications of pulmonary tuberculosis which is almost always associated with pleural space infection [2]. However, more commonly it arises as a complication of lung resection surgery following the failure of the bronchial stump to heal, which may be from improper initial closure, inadequate blood supply, infection at the bronchial stump, or residual malignant tumour at the bronchial stump [1, 3]. Its incidence is reported to be 4–20% after pneumonectomy and < 1% after Lobectomy [4] and it is 4 to 5 times more likely after right pneumonectomy as compared with left pneumonectomy [5, 6].

The risk factors for the development of BPF in the setting of anatomic lung resection include the extent of lung resection, preoperative radiotherapy, prolonged postoperative ventilator support, adult respiratory distress syndrome, chronic obstructive pulmonary disease, poor nutrition, steroids, and diabetes [7–9].

The clinical presentation of a BPF can be acute with sudden expectoration of purulent sputum, cough, dyspnea, mediastinal and tracheal shifts, subcutaneous emphysema, and persistent post-operative air leak if the chest tube is in place [2, 7, 8, 10].

In postpneumonectomy patients with BPF, an acute decrease in the pleural fluid level or disappearance of pleural fluid is seen on X-ray. The subacute and chronic clinical presentations of BPF are usually associated with an infected pleural space and present with more insidious symptoms with productive cough, fever, leukocytosis and a new air-fluid level appears in a previously opacified hemothorax [1, 5, 11]. Computed tomography, may reveal pneumothorax, pneumomediastinum, and underlying lung pathology and may demonstrate actual fistulous communication in a subset of patients. Fiberoptic bronchoscopy (FOB) has been used to localize/confirm BPF [1, 5].

The initial management of patients with BPF must include drainage of the infected pleural space, antibiotics and quick diagnosis and early treatment of underlying tuberculosis. The difficulty in the management of BPF remains to close the fistula and manage the residual space; once the infection is controlled [12, 13].

The choice of treatment depends on the patient’s functional status, duration of BPF, and its size and clinical features. Different surgical techniques have been used including debridement and closure with buttressing of a pedicled omental or intrathoracic muscle transposition and completion pneumonectomy to aggressive surgical options such as open-window thoracostomy, thoracoplasty and a trans-sternal mediastinal approach which is problematic for an already compromised patient. Biologic glues have been applied bronchoscopically to achieve endobronchial closure of BPF in several case reports [1, 5, 14, 15].

Bronchopleural fistulas constitute a major therapeutic challenge for the thoracic surgeon. Hospitalization is long, multiple operative procedures often are necessary and morbidity is high. The presence of a BPF is an independent predictor of mortality and the most frequent cause of death is pneumonia in the contralateral lung. BPFs complicating lung resections are considered early if they occur within 30 days of surgery, which usually carries a higher mortality rate. The reported mortality rates associated with early BPF are 11.6–18% and 0–7.1%with late BPF [10, 16–18].

To the best of the researcher’s knowledge, there is no study done on the assessment of risk factors and treatment outcomes of bronchopleural fistula in Ethiopia so far. So, our study will help us to assess the magnitude of the problem, risk factors, type of treatment given and their outcome which help us to suggest preventive measures and treatment options which lead to better outcomes for patients with BPF.

## Method and material

### Study design and setting

An institution-based multicenter 10 years retrospective cohort study was conducted at Tikur Anbessa Specialized Hospital (TASH), Menelik the 2nd Referral Hospital, and Teklearegay Zenebech Negest Aster (TZNA) General Hospital from September 13 to September 30, 2022. TASH is the teaching hospital of Addis Ababa University (AAU) and offers diagnosis and treatment for approximately 370,000-400,000 patients a year with a capacity of over 800 beds and 27 surgical beds. Menelik the 2nd referral hospital is one of the oldest public hospitals in the country with a surgical bed capacity of 135 and TZNA general hospital is a private hospital with 27 beds. The hospitals provide cardiothoracic surgical services in addition to other routine services.

### Study participants recruitment

A total of 52 patients’ cards with the diagnosis of bronchopleural fistula were selected for the study. It was a Survey of Data of all patients who have been admitted and treated at Tikur Anbessa, Menelik the 2nd referral hospital, and TZNA general hospital in the cardiothoracic unit from September 2012- August 2021.

### Data collection

Review of records of patients using a data extraction checklist which was developed after a review of the literature [5, 6, 10] was used to extract data on the socio-demographic characteristics of the patients and clinical information, types and indication of surgery, distribution of post-operative technical factors, treatment given (types of conservative management), types of surgical management and complications are used as independent variables and magnitude of bronchopleural fistula, risk factors and treatment outcomes are used as dependent variables in this study. To assure the quality of data collection, one day of training was given to data collectors and supervisors on the objectives of the study, data collection tools, and research ethics. The data was retracted by 3 general practitioners and one supervisor of a Master of Public Health (MPH) holder.

### Data analysis

The data were entered into Epi data (Manager and Entry client) 4.6 version statistical software and the generated data was exported to SPSS version 25 for analysis. Descriptive statistics were used. The mean and standard deviation (SD) were calculated for the continuous variables and -cross-tabulation analysis was used to see the relationship between variables.

## Result

The data was retrieved from three hospitals found in Addis Ababa, 30 (57.7%) were from black lion specialized hospital, 19(36.5%) from Menelik the 2nd referral hospital and 3(5.8%) from TZNA general hospital.

The prevalence of bronchopleural fistula was 2% (no = 52) with 95% CL (1.72, 2.28) from 2546 patients admitted to the cardiothoracic unit in the three hospitals studied in the past ten years. From the total 52 patient cards reviewed, 36 (69%) of study participants were male and the mean age was 33.42 with SD = 12.5.

Most study participants 69.2% (n = 36) were diagnosed to have tuberculosis and 1(1.9%) is diagnosed with the coronavirus. From a total reviewed card, 15 patients’ cards were on anti TB at diagnoses of bronchopleural fistula (BPF); however, nobody was diagnosed with necrotized pneumonia. On the assessment of risk factors only Six (11.5%) of study participants were diagnosed to have a fungal infection, 1 (1.9%) with diabetes, and 2 (3.8%) had a history of steroid use. The most common clinical presentation was productive cough present in 49 (96.2%) cases, 48(92.3%) cases had shortness of breath, 39 (75%) had a fever, 6 (11.5%) cases has pus draining from the chest wall and only 3 (5.8%) had respiratory distress (Table 1).

**Table 1:** Clinical characteristics of the study participants of the prevalence and Treatment Outcome of Bronchopleural Fistula

Clinical characteristics		Frequency	Percent
Diagnosed to have tuberculosis?	yes	36	69.2
	No	16	30.8
Diagnosed to have a fungal infection	yes	6	11.5
	No	46	88.5
History of COVID-19?	Yes	1	1.9
	No	51	98.1
Diagnosed to have Diabetes mellitus	Yes	1	1.9
	No	51	98.1
People with HIV	Yes	1	1.9
	No	51	98.1
History of Steroid Use	Yes	2	3.8
	Unknown	50	96.2
Productive Cough	Yes	49	94.2
	No	3	5.8
Shortness of breath	Yes	48	92.3
	No	13	25.0
History of Fever	Yes	39	75.0
	No	4	7.7
Pus draining from the chest wall	Yes	6	11.5
	No	49	94.2
Respiratory distress at diagnosis?	Yes	3	5.8
	No	46	88.5
History of thoracic surgery	Yes	20	38.5
	No	51	98.1
History of thoracic trauma	Yes	1	1.9
	No	32	61.5

When we see radiologic investigations, 46 (88.5%) cases underwent an X-ray examination and air-fluid levels were found in 33 (63.4%), and 1 (1.9%) has features of tension pneumothorax. CT scan was done for thirty-four (65.4%) cases and out of them 7 (13.5%) showed bronchopleural communication, and bronchoscopy was done only for one patient which showed a small hole in the right post-pneumonectomy bronchial stump communicating to the pleura.

**Fig. 1:**
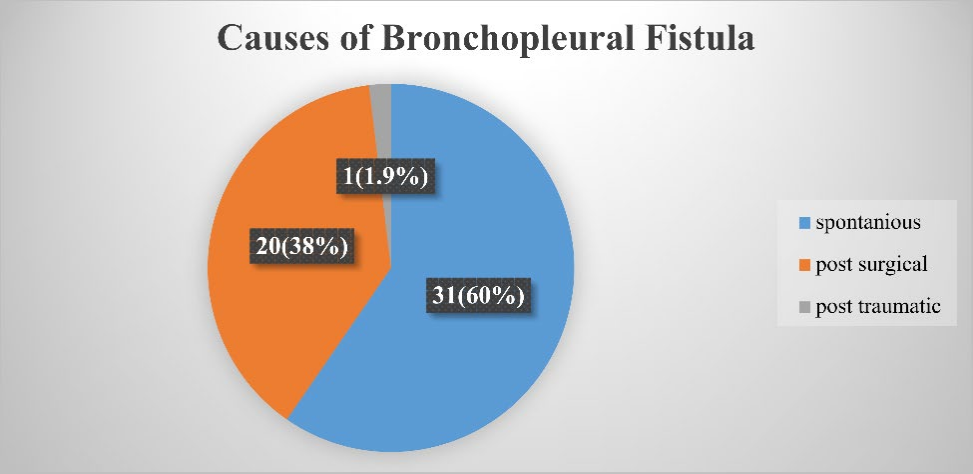
Causes of bronchopleural fistula of study participants of the Prevalence and Treatment Outcome of Bronchopleural Fistula

From the total of 52 reviewed cases with the diagnosis of BPF 31 (60%) of them were spontaneously developed and 20 (38%) were post-surgical. Of- the postsurgical BPFs 12(60%) of them were diagnosed to have tuberculosis and 1(1.9%) was a post-traumatic fistula (Fig. 1). From the spontaneously developed fistulas 24 (77.4%) cases were post-tuberculosis and one (3%) was post COVID-19.

Of the total of 20 (38%) post-surgical bronchopleural fistula, 14 (26.9%) of them were lung resection followed by hydatid cystectomy 4 (7.7%) and the remaining are decortications, and bullectomy accounts for 1(1.9%) each respectively. The Indication for lung resection was infectious in 12(85.7%) (Bronchiectasis, aspergilloma, post-TB fibrotic lung and hydatid cyst) cases and 2 (14.3%) was non-infectious (lung cancer, bullae) cases Table 2).

**Table 2:** The types & indications of surgery of study participants of the Prevalence and Treatment Outcome of Bronchopleural Fistula

Type of surgical management and indication for lung resection		Frequency	Per cent
Type of surgery	Lung resection	14	26.9
	Hydatid cystectomy	4	7.7
	Decortication	1	1.9
	Bullectomy	1	1.9
Indication for lung resection	Bronchiectasis	3	21.5
	Post TB, Aspergilloma with fibrotic destroyed lung	3	21.5
	Hydatid cyst	2	14.3
	Massive hemoptysis with bronchiectasis	2	14.3
	Bronchiectasis plus aspergilloma	2	14.3
	Right upper lobe bullae	1	7.1
	Lung cancer	1	7.1

**Fig. 2:**
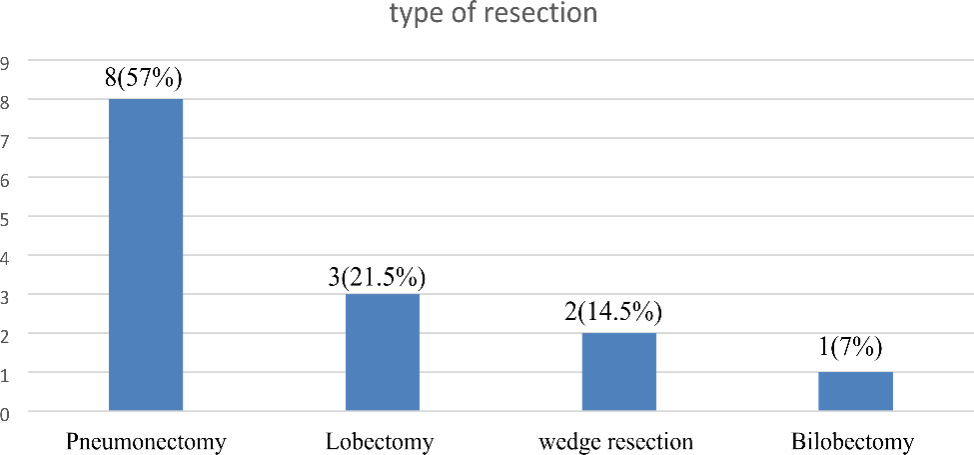
Type of resection for post resectional BPF of study participants of the Prevalence and Treatment Outcome of Bronchopleural Fistula

From the total of 14 lung resections, the duration from the time of lung resection to a diagnosis of the bronchopleural fistula was early (less than one month) in 8 (57%) and late (greater than one month) in 6 (43%). Concerning the type of resection 8 (57%) were pneumonectomy of these 4(50%) were right pneumonectomy and the remaining were 3 (21.5%) Lobectomy, 2 (14.5%) wedge resection and 1(7%) bilobectomy respectively (Fig. 2).

Only two (14.3%) of the cases with lung resection used a flap for coverage of bronchial stump closure. Regarding the stitch used for bronchial closure, prolene 9 (71.5%) was used more frequently followed by silk 2 (14.5%). The most commonly used technique for bronchial stump closure was continuous two layers 13 (93%). Of the cases having thoracic surgical history only one (5%) patient has used postoperative mechanical ventilation and 7 (35%) cases have prolonged air leaks (Table 3).

**Table 3:** The distribution of post-operative and technical factors of lung resection for post-resection BPF of study participants of the Prevalence and Treatment Outcome of Bronchopleural Fistula

Post-operative and technical factors		Frequency	Per cent
Coverage of stump with flap	Yes	2	14.3
	No	12	85.7
Type of flap	Intercostal muscle	1	50
	Pleural flap	1	50
Type of stitch used	Prolene	10	71.5
	Silk	2	14.5
	Stapler	1	7
	Vicryl	1	7
The technique of bronchial stump closure	Continuous two layers	13	93
	Stapler	1	7
Patient on a mechanical ventilator postoperatively	yes	1	5
	No	19	95
Prolonged air leak	yes	7	35
	No	13	65

**Fig. 3:**
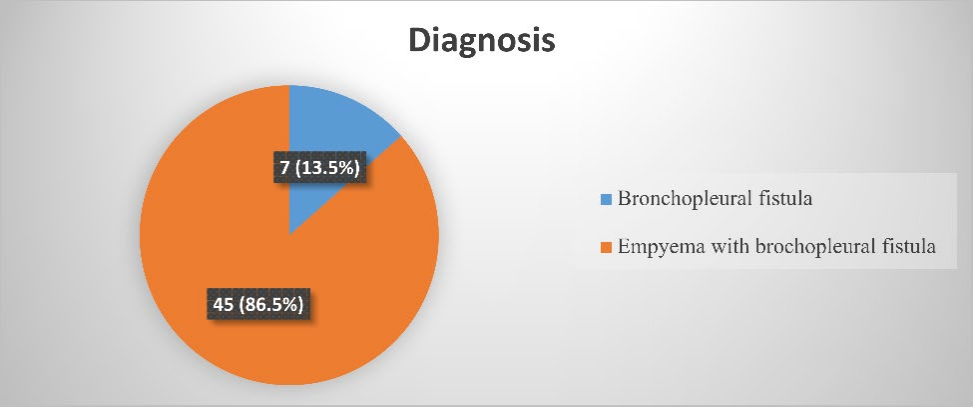
Types of diagnosis of study the participants of prevalence and treatment outcome of broncho pleural fistula

When we come to the diagnosis, most of the patients (45(86.5%)) have a diagnosis of empyema with bronchopleural fistula, and 7 (13.5%) of cases were diagnosed to have a bronchopleural fistula (BPF) only (Fig. 3).

Concerning management, 32 (61.5%) were managed surgically and 20 (38.5%) were managed conservatively. Of the conservative treatment, 12 (60%) were tube thoracostomy and observation, 7(35%) were tube thoracostomy with medical management and 1(5%) observation with medical management. When we see the outcome of conservative management, 18 (90%) of them had a failure of conservative management and changed to surgical management and in one (5%) the BPF was closed but the other was still on follow-up (Table 4).

**Table 4:** Treatment given, type of conservative management and outcome of conservative management of study participants of the prevalence and Treatment Outcome of Bronchopleural Fistula

Treatment given, type of conservative management and outcome of conservative management of study participants		Frequency	Per cent
Treatment given	Surgical	32	61.53
	Conservative	20	38.5
Type of conservative management	Tube thoracostomy and observation	12	60
	Tube thoracostomy and medical management	7	35
	Observation with medical management	1	5
The outcome of conservative management	Failure of conservative management and change to surgical	18	90
	Still on conservative management	1	5
	Closure of BPF	1	5

A total of 65 surgeries were done for 50 cases managed surgically, and the most common procedure done was decortication and bronchial hole closure 21 (40.4%) followed by open window thoracotomy 13 (25%). Of the total surgically managed cases, a flap was used for seven (22%) and the most common flap used was latissimus dorsi 3(43%) (Table 5).

**Table 5:** The type of surgical management and type of flap used in study participants of the prevalence and Treatment Outcome of Bronchopleural Fistula

Type of surgical management and flap used		Frequency	Per cent
The type of surgical management	Decortication and bronchial hole closure	21	40.4
	Open window thoracostomy	13	25.0
	Closure of bronchial hole	7	13.5
	Pneumonectomy	6	11.5
	Thoracoplasty	5	9.6
	Open window thoracostomy and bronchial hole closure with muscle flap	4	7.6
	Decortication and closure of bronchial hole with flap	2	3.8
	Others ***	7	13.3
	Total	65	100
kind of flap used	Latissimus dorsi muscle	3	37.5
	Intercostal muscle	2	25
	Mediastinal pleural	2	25
	Latissimus dorsi & intercostal muscle	1	12.5
	Total	8	100

Others *** multiple surgeries (bronchial hole closure with mediastinal pleural flap, decortication + left apico-posterior pleurectomy, evacuation of pus, right lower lobectomy plus decortication, right upper lobe wedge resection, and with combinations of the above surgeries).

The mean number of surgeries done was 1.3 + 0.614 with a range of 1 to 4 surgeries. Out of 50 surgically managed cases, 12 (23.1%) of them has complications during their hospital stay, and the most common complication was pneumonia 10 (66.6%) of these three cases have additional complications. The median duration of hospital stay was 13 + 25.376 days with a range of 2 to 160 days (Table 6).

**Table 6:** Complications during hospital stay of study participants of the Prevalence and Treatment Outcome of Bronchopleural Fistula

Complications during a hospital stay		Frequency	Per cent
Complications during hospital stays	yes	12	23
	No	40	77
Complications	Pneumonia	10	66.6
	Prolonged air leak	2	13.3
	Decompensated liver disease	1	6.7
	Non-expanded lung	1	6.7
	Post pneumonectomy space empyema	1	6.7

**Fig. 4:**
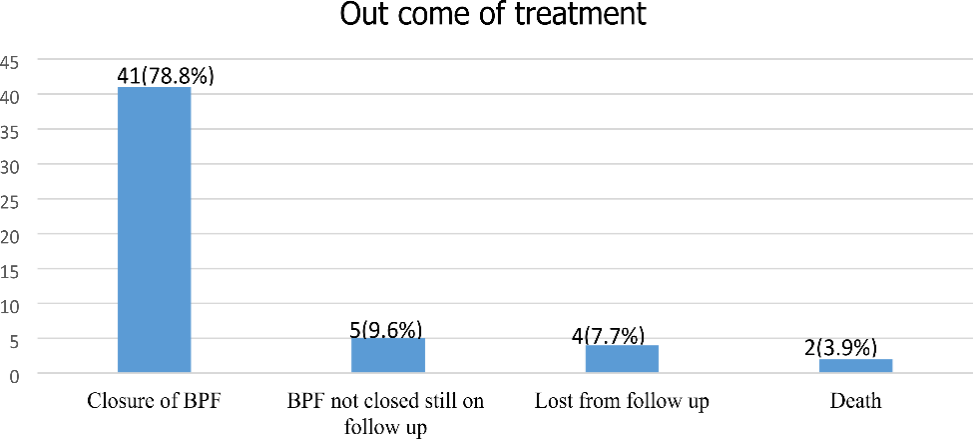
Treatment outcome of study participants of prevalence and treatment outcome of broncho pleural fistula

Of the 52 cases reviewed bronchopleural fistula (BPF) closed in 41 (78.8%) and 40 cases were managed surgically and one case was managed conservatively, in 5(9.6%) cases BPF was not closed and was still on follow-up. There were two (3.9%) deaths, and the cause of death was sepsis secondary to pneumonia of the contralateral lung in one and unknown for the other case. Four cases were lost from follow-up, and five developed recurrent fistulas (Fig. 4).

Out of 14 post-lung resections BPF 8 (57%) were early and out of them fistula was closed in 6 cases and one (7%) died in early post-operative BPF (Table 7).

**Table 7:** Treatment outcome according to the duration of resection to the diagnosis of BPF of the Prevalence and Treatment Outcome of Bronchopleural Fistula

Duration from resection to the diagnosis of BPF		the outcome of the treatment				Total
		Closure of BPF	BPF is not closed still on follow up	Death	Lost from follow up	
	<=30 days	6	1	1(7%)	0	8(57%)
	> 30 days	4	1	0	1	6(43%)
Total		10	2	1	1	14(100%)

The most common type of surgical management was decortication and bronchial hole closure in 21 cases and out of them 19 led to the closure of the fistula but only in one BPF not closed and still on follow-up. The second most common procedure done was open window thoracostomy which is done in 13 patients out of whom 6 had their BPF closed and in 3 BPF not closed and still on follow-up (Table 8).

**Table 8:** Treatment outcome according to the type of surgical management of study participants of the Prevalence and Treatment Outcome of Bronchopleural Fistula

The type of surgical management	Outcome of treatment				Total
	Closure of BPF	BPF is not closed still on follow up	Death	Lost from follow up	
Decortication and bronchial hole closure	19	1	1	0	21
Open window thoracostomy	6	3	1	3	13
Closure of bronchial hole	7	0	0	0	7
Pneumonectomy	4	0	0	1	5
Decortication closure of bronchial hole and flap	2	0	0	0	2
Open window thoracostomy and bronchial hole closure with muscle flap	2	0	0	0	2
Total	40	4	2	4	50

The mean duration of BPF closure from the time of surgery was 82.41 + 123.396 days which ranges from one day to 380 days. For cases who are still on follow-up, the mean duration from the time of surgery till the time of data collection was 632.5 + 460.561 ranging from 107 days to 1097 days.

sa a.

## Discussion

Spontaneous BPF is more common in our study though currently complications during bronchopulmonary procedures are the leading cause of BPF [19] the possible reason might be a high prevalence of tuberculosis and a low number of lung resections done in our set-up. A bronchopleural fistula may result from necrotizing pneumonia/empyema, lung neoplasm, and blunt and penetrating lung injuries may occur as a complication of procedures or may complicate radiation therapy [2]. [

Bronchopleural fistula is seen as a potentially catastrophic complication of pulmonary tuberculosis, which is still endemic in the developing world [13] in our study 69.2% of the study participants were diagnosed to have tuberculosis. This is comparable to one study done at the Maryland School of Medicine, a series of 77 patients treated for bronchopleural fistula over − 13 years [18].

Post-pneumonectomy BPF was more common than post-lobectomy BPF in our study which is comparable with a study done in Italy on the incidence and management of post-lobectomy and pneumonectomy bronchopleural fistula [4].

Bronchopleural fistula is more common after right pneumonectomy than the left, in one study done on the risk of right pneumonectomy on 187 patients with pneumonectomy [6] also in another study done in Japan [20] which is not comparable with our study where the post pneumonectomy cases 50% are right side, the possible reason is most of the indications for lung resection in our study were infectious causes (85.7%) leading to accumulation of secretions in the left bronchus which is longer than right, unlike the study where the most common indication was lung cancer (98.5%).

Most BPFs occur early (within one month) after lung resection in post-resectional BPF cases in our study and carry a higher mortality rate (7%). This is comparable to a study done in China, out of 6,239 lung resections for non-small cell cancers there were 23 late and 43 early bronchopleural fistulas and the mortality rate of the early bronchopleural fistula was 11.6%, which was significantly higher Compared with the mortality rate of late bronchopleural fistula 0% [17].

The diagnosis of bronchopleural fistula must be suspected when there is a persistent post-operative air leak [1], which occurred in 5 patients out of 17 patients who had post-operative bronchopleural fistula in one study [14]. This is comparable to our study where seven (35%) of the cases have prolonged postoperative air leaks. 

Appropriate management of a BPF depends on the type of fistula and the clinical condition of the patient [5]. In the developing world which is endemic for tuberculosis, the resolution of bronchopleural fistula depends on quick diagnosis and early treatment of underlying tuberculosis, and careful management of intercostal drainage. In a case report of a 55 years old patient with spontaneous bronchopleural fistula diagnosed to have tuberculosis which was managed with intercostal drain and anti-TB which lead to the closure of BPF [13] Which is Similar to our study where two patients managed conservatively and both were diagnosed to have tuberculosis and managed with anti-TB, one spontaneously developed BPF which is still on follow up and the other was post pneumonectomy BPF in which fistula was closed.

Surgery was needed for most of the patients in our study and the most common type of surgical management was decortication and bronchial hole closure which leads to closure of the fistula in most of the cases which are comparable to a study done on management options for tuberculous bronchopleural fistula where decortication with BPF repair was done in most patients surgically managed which affected closure in all patients [2]. The Second most common procedure done was open window thoracostomy which leads to closure in most but in 3 cases BPF was not closed and still on follow-up with a mean duration from the time of surgery till the time of data collection of 632.5 ± 460.561 days on prolonged wound care. Similar to other literature where an open window thoracostomy is still an option for the management of BPF [21, 22]There is some controversy on the timing for closing the window, but in most cases, closure is warranted ~ 6 months after thoracostomy [5, 22].

The most common cause of death is aspiration pneumonia with subsequent acute respiratory distress syndrome as occurred in one (5.8%) patient out of 17 post-surgical BPFs in one study [14] which is comparative in our study, there were 2 (3.9%) deaths and the cause of death was sepsis secondary to pneumonia of contralateral lung in one and unknown for the other case.

### Strength and limitation

The study was relayed on a retrospective study because of the limited number of patients. So, using secondary data affects the quality of information. A retrospective study generally only establishes association and not cause-effect between risk factors and outcome. Despite these limitations, this study was the first of its kind to be conducted in Ethiopia.

## Conclusion

Broncho pleural fistula is common in patients with a diagnosis of tuberculosis occurring both spontaneously and after lung resection.

Undergoing thoracic surgery, especially lung resection is one Of the leading risk factor for the development of bronchopleural fistula and BPF is more common after pneumonectomy than other types of lung resection. Diagnosis of BPF is common in patients having prolonged postoperative air leak.

Surgical management is needed for most patients with a diagnosis of bronchopleural fistula. Management with open window thoracostomy needs prolonged wound care.

### Recommendations

We should have a high index of suspicion for a diagnosis of BPF in patients having prolonged postoperative air leak and patients presenting with product cough after pneumonectomy in patients with a diagnosis of tuberculosis. Further workup should be done and the patient managed accordingly.

Management of bronchopleural fistula needs to be individualized, dictated by the initial response. Open window thoracostomy should be reserved for patients who cannot tolerate major surgery and after failure of other management options, and pleural space should be drained immediately after the diagnosis of Bronchopleural fistula is made to prevent contamination of the contralateral lung. Future research should be done on bronchopleural fistula and be prospective using longitudinal studies.

### Definitions of terms and operational definition

#### Air leak (alveolar - pleural fistula)

is a communication between the pulmonary parenchyma distal to a segmental bronchus and the pleural space [11].

#### Bilobectomy

Resection of two lobes of a lung [11].

#### Bronchopleural fistula

is a communication between the main, lobar or segmental bronchus and pleural space [10].

#### BPF closed

when signs and symptoms of BPF are absent no air-fluid level on x-ray and the chest wound is healed.

#### Lobectomy

Resection of one lobe of a lung [11].

#### Open window thoracotomy

Removing a portion of two to three ribs and undermining the subcutaneous tissues circumferentially so that they can be sutured down to the end thoracic fascia [10].

#### Pneumonectomy

Resection of one lung [11].

#### Prolonged air leak

an air leak beyond the fifth postoperative day [11].

#### Spontaneous BPF

BPF in patients having no history of trauma or surgery.

TASH: Tikur Anbessa Specialized Hospital.

TZNA: Teklearegay Zenebech Negest Aster.

#### Wedge resection

Resection of part of a segment of a lung [11].

## Data Availability

Data will be available on request.
